# Universal parameters of bulk-solvent masks

**DOI:** 10.1107/S2053273324000299

**Published:** 2024-02-09

**Authors:** Alexandre Urzhumtsev, Paul Adams, Pavel Afonine

**Affiliations:** a Centre for Integrative Biology, Institut de Génétique et de Biologie Moléculaire et Cellulaire, CNRS–INSERM-UdS, 1 rue Laurent Fries, BP 10142, 67404 Illkirch, France; b Faculté des Sciences et Technologies, Université de Lorraine, BP 239, 54506 Vandoeuvre-les-Nancy, France; c Molecular Biophysics and Integrated Bioimaging Division, Lawrence Berkeley National Laboratory, Berkeley, California, USA; d Department of Bioengineering, University of California, Berkeley, Berkeley, California, USA; University of Warsaw, Poland

**Keywords:** bulk-solvent modelling, flat models, solvent masks, grid steps, mask parameters

## Abstract

The bulk solvent is a major component of biomacromolecular crystals that contributes significantly to the observed diffraction intensities. Accurate modelling of the bulk solvent has been recognized as important for many crystallographic calculations. Owing to its simplicity and modelling power, the flat (mask-based) bulk-solvent model is used by most modern crystallographic software packages to account for disordered solvent. In this model, the bulk-solvent contribution is defined by a binary mask and a scale (scattering) function. The mask is calculated on a regular grid using the atomic model coordinates and their chemical types. The grid step and two radii, solvent and shrinkage, are the three parameters that govern the mask calculation. They are highly correlated and their choice is a compromise between the computer time needed to calculate the mask and the accuracy of the mask. It is demonstrated here that this choice can be optimized using a unique value of 0.6 Å for the grid step irrespective of the data resolution, and the radii values adjusted correspondingly. The improved values were tested on a large sample of Protein Data Bank entries derived from X-ray diffraction data and are now used in the computational crystallography toolbox (*CCTBX*) and in *Phenix* as the default choice.

## Abstract 2

The optimal choice of bulk-solvent mask parameters (grid step, and solvent and shrinkage radii) has been revised.

## 1. Introduction

Bulk solvent (or disordered solvent) on average occupies about half the volume of a macromolecular crystal and noticeably contributes to the medium- and low-resolution structure factor intensities [see *e.g.* Weichenberger *et al.* (2015 ▸) for a recent review]. It is therefore important to include its contribution into the model-calculated structure factors to account for the entire unit-cell contents adequately. The procedure needs to be fast and accurate because this calculation is repeated many times during atomic model refinement.

The flat (or mask-based) bulk-solvent model (Jiang & Brünger, 1994 ▸) is currently the option of choice in most crystallographic software packages. The model first requires the definition of a solvent mask in the unit cell. This mask is a binary function calculated on a regular grid with values of zero inside the molecular region and one outside. The Fourier coefficients **F**_mask_(**s**) of this binary mask are then calculated and scaled together with the structure factors **F**_calc_(**s**) calculated from the atomic model,

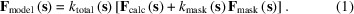

The resolution-dependent scales *k*_mask_(**s**) and *k*_total_(**s**) are obtained by fitting **F**_model_(**s**) to the experimental data [see *e.g.* Afonine *et al.* (2013 ▸)].

The mask calculation as introduced by Jiang & Brünger (1994 ▸) uses the following parameters:

(i) The size of the grid step *d*_grid_.

(ii) The solvent probe radius *r*_probe_ or *r*_solv_.

(iii) The shrinking radius *r*_shrink_.

(iv) Tabulated atomic van der Waals radii.

The mask calculation procedure involves augmenting the atomic van der Waals radius with the solvent probe radius to create a sphere of combined radius around each atom. Grid points falling outside of these spheres, which define the expanded macromolecular region, are designated as the solvent-accessible region surrounding the macromolecule. Subsequently, all grid points within a distance *r*_shrink_ from any point of the tentative solvent-accessible region defined above are assigned to the bulk-solvent region. The resulting mask is referred to as the bulk-solvent mask.

An optimal choice for these parameters should balance structure factor accuracy and the time required to compute the mask and its Fourier coefficients by Fourier transformation. Based on two cases at 2.2 and 1.8 Å resolution, Jiang & Brünger (1994 ▸) determined optimal choices for *r*_probe_, *r*_shrink_ and *d*_step_ to be 1.0, 1.1 and (*d*_min_/4) Å, respectively, where *d*_min_ is the resolution of the data set. Later, Rees *et al.* (2005 ▸) showed that for low-resolution data sets a step size of (*d*_min_/4) Å is too coarse, leading to inaccurate masks, and that a step size somewhere between *d*_min_/5 and *d*_min_/10 is more appropriate. While this solves the problem of mask accuracy at low resolution, at high resolution such a fine grid step will result in a significant (and unnecessary) increase in computational time.

In this work, we suggest that the grid step for mask calculation should not depend on the resolution. We demonstrate that using a step size of 0.6 Å, along with values of *r*_solv_ and *r*_shrink_ set to 1.1 and 0.9 Å, respectively, does not compromise the accuracy of the mask or the calculation time. Therefore, we recommend this combination of parameters for calculating the bulk-solvent mask for structures of any resolution.

## 2. Method

### 2.1. Why is a common resolution-independent grid step expected?

The electron-density distribution of a macromolecule in a crystal is a peaky function, while the function that describes the bulk solvent is a flat function with a smooth border [see *e.g.* Fenn *et al.* (2010 ▸)]. Consequently, the Fourier coefficients that describe the bulk-solvent distribution decrease sharply, and usually become negligibly small, at resolutions better than *d*_solv_ ≃ 3.5–4.0 Å [see *e.g.* Phillips (1980 ▸), Jiang & Brünger (1994 ▸) or Afonine *et al.* (2013 ▸)]. To understand the consequences of this on the choice of the grid step, we refer to a one-dimensional example below.

For a periodic function of a single variable with period *a*, the integral Fourier transform gives an infinite number of Fourier coefficients **F**(*h*), where *h* is an integer, both positive and negative. When such a function is sampled on a regular grid with *N* points per interval, the discrete Fourier transform yields only *N* independent values **F**_grid_(*h*), *e.g.*


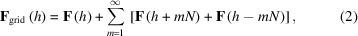

for −*N*/2 < *h* ≤ *N*/2 [see, for example, formula (4) in Ten Eyck (1977 ▸)]. **F**_grid_(*h*) differ from the respective **F**(*h*) by a convergent infinite series of correcting terms (Appendix *A* [App appa]) where 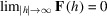 . Let us suppose that **F**(*h*) values are equal exactly to zero for |*h*| > *H* = *a*/*d*_solv_ with some resolution limit *d*_solv_. If *N* in (2) is sufficiently large, for example, *N* > 2*H*, all correcting terms with indices *h* ± *mN* are also zero, resulting in **F**_grid_(*h*) = **F**(*h*) as desired. Taking *N* larger than 2*H*, *i.e.* taking the grid step *d*_grid_ = *a*/*N* smaller than

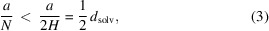

has no effect. Conversely, taking smaller *N* makes **F**_grid_(*h*) different from **F**(*h*) by at least one non-zero term **F**(*h* ± *mN*), *m* ≠ 0. The analogue of (2) for three-dimensional functions can be found in Sayre (1951 ▸), Lunin *et al.* (2002 ▸), Navaza (2002 ▸) and Afonine & Urzhumtsev (2004 ▸).

This suggests the potential existence of a universally optimal grid step 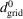 for the problem under study, which is related to *d*_solv_ ≃ 3.5 Å in a manner similar to (3), albeit with a scale factor that may not be equal to 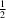 ; the latter arises from the facts that these high-resolution structure factors may be different from exactly zero and the Fourier analysis is carried out in three-dimensional space.

### 2.2. Models and data

The search for the optimal bulk-solvent mask parameters was conducted using 277 quality-filtered models and X-ray diffraction data obtained from the Protein Data Bank (PDB; Burley *et al.*, 2021 ▸). The quality filters included a crystallographic *R* factor better than 0.25, overall and per-resolution shell data completeness above 95%, no data pathologies such as twinning, and a relatively high upper data resolution limit (*d*_min_ ≤ 3.0 Å). To accelerate the calculations, we excluded very large models. The results obtained with these 277 models were then validated with a larger set of 2077 structures used in a previous bulk-solvent study (Afonine *et al.*, 2013 ▸) and representing a broad range of model sizes and data resolutions, from subatomic to low.

Among the 277 models, 54% were refined using *Refmac* (Murshudov *et al.*, 2011 ▸), 25% using *Phenix* (Afonine *et al.*, 2012 ▸), 14% using *CNS/X-plor* (Brünger *et al.*, 1998 ▸), 0.04% using *BUSTER*/*TNT* (Tronrud *et al.*, 1987 ▸; Roversi *et al.*, 2000 ▸; Blanc *et al.*, 2004 ▸) and 0.03% using *SHELX* (Sheldrick, 2015 ▸). These programs may potentially employ different types of bulk-solvent models, for example the Babinet-based model (Langridge *et al.*, 1960 ▸; Moews & Kretsinger, 1975 ▸) in *SHELX*, *Refmac* and *BUSTER*/*TNT*, as well as different parameters for mask calculations when the mask-based solvent model was used, such as in *Phenix*, *CNS/X-plor* and again *Refmac*. We believe that this diversity in refinement programs, each with its distinct formulation of bulk-solvent modelling, helps mitigate any potential model bias related to the solvent parameters used in these programs.

### 2.3. Finding optimal values for *r*_solv_, *r*_shrink_ and *d*_grid_

For each selected PDB entry, the values of *d*_grid_ were systematically sampled in the range between 0.2 and 1 Å in steps of 0.1 Å, and both *r*_solv_ and *r*_shrink_ were sampled between 0.5 and 1.5 Å, also in steps of 0.1 Å. For each trial triplet of values (*r*_solv_, *r*_shrink_, *d*_grid_), the scales *k*_total_(**s**) and *k*_mask_(**s**) in (1) were re-calculated as detailed by Afonine *et al.* (2013 ▸), and the *R*-factor values, referred to as *R* ^(4)^, were calculated using all reflections up to 4 Å resolution. In what follows, 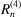 stands for *R* ^(4)^ calculated for the structure numbered *n*. These values were the principal information to identify potential universal values for 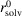 , 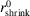 and 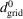 . The details follow in Section 3.

## 3. Results

### 3.1. Variation of the optimal *R* factor with the mask grid step

The search for common parameters was based on the hypothesis that there is a common behaviour of 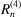 with parameter values for different structures. First, we tried to decouple the search for optimal *d*_grid_ and (*r*_solv_, *r*_shrink_). This was achieved by finding the combination of (*r*_solv_, *r*_shrink_) that minimizes 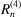 for each trial *d*_grid_ and for each structure *n*,

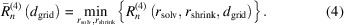

Obviously, these values are different for each structure, but they vary with *d*_grid_ very similarly. In particular, this is true for their variation around the average of 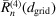 over grid steps

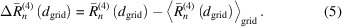




**Figure 1:**
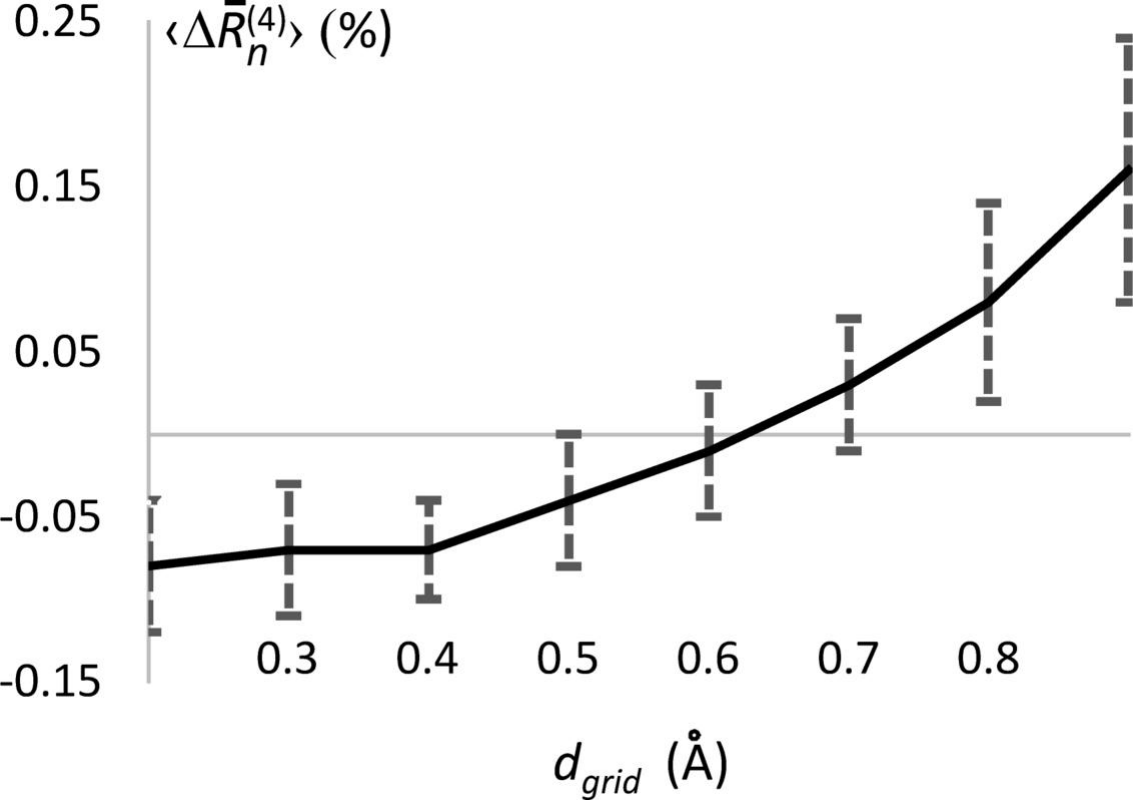
The variation 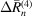 in the 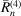 factor, as defined in the text, as a function of the grid step *d*_grid_. Each data point is the average of 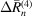 across all structures. Intervals of 1σ are given for each grid step.

Subtracting the average in (5) makes the dependency of 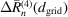 on *d*_grid_ similar for all structures, which in turn makes it possible to analyse the average of these dependencies over all structures (Fig. 1 ▸).

It is to be expected that 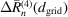 should increase with step size. However, the value does not change significantly for *d*_grid_ in the range of 0.2–0.4 Å, suggesting that steps smaller than 0.4 Å are unnecessarily small. Above this step value, 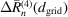 starts to increase, and the goal is to find a compromise between the introduced errors and the gain in computation time. Increasing the step from 0.4 Å to 0.6 Å or 0.8 Å increases the grid size, and therefore the number of computing operations, by about four times or eight times, respectively.

A step size of 1.0 Å resulted in very large errors and was excluded from further analysis. Calculations with a step size of 0.9 Å resulted in a large number of outliers with large 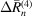 , making this step size also unsuitable. This leads to 0.4–0.8 Å as the range for the grid-step search.

### 3.2. Acceptable combinations of parameters

Next, for each model *n*, we analysed the parameter values that lead to the lowest 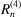 value across all combinations of (*r*_solv_, *r*_shrink_, *d*_grid_),

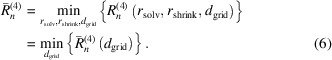




It is possible that, for a given structure, several combinations of (*r*_solv_, *r*_shrink_, *d*_grid_) result in 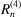 values that are close to the global minimum of (6). To address such small fluctuations in the 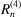 values, we introduce a parameter ɛ_*R*_ considering all values 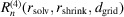 ≤ 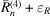 to be as good as 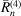 , where the value of ɛ_*R*_ varies in the range 0.001–0.002.

The parameter values (*r*_solv_, *r*_shrink_, *d*_grid_) corresponding to (6) are expected to vary from one structure to another, and we are looking for the combinations that are persistent over all structures. As a formal quantitative measure, for each set of parameters (*r*_solv_, *r*_shrink_, *d*_grid_) and for each structure *n*, we calculate a non-negative value

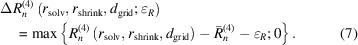




To be able to combine the distribution of (*r*_solv_, *r*_shrink_, *d*_grid_) for each structure into one cumulative distribution over all structures, we convert 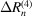 in (7) into

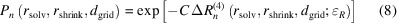

with constants *C* > 0 and ɛ_*R*_. The product of (8) over all structures,

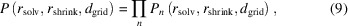

reflects both the contrast of the lowest 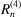 for an individual structure and the persistence of the parameter values over all structures. *P*(*r*_solv_, *r*_shrink_, *d*_grid_) varies from 0 to 1; the higher the value of (9), the more preferable the combination of parameters. Calculations with data sets of different sizes suggested the choice of *C* in the range between 0.01 and 1.0 and ɛ_*R*_ as stated above in order to obtain a decent contrast while keeping points with neighbouring 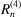 values. In general, we observed that the variation in the constants *C* and ɛ_*R*_ around the values given above obviously modifies the contrast of the distribution (9) while not influencing the location of its peaks.

**Figure 2:**
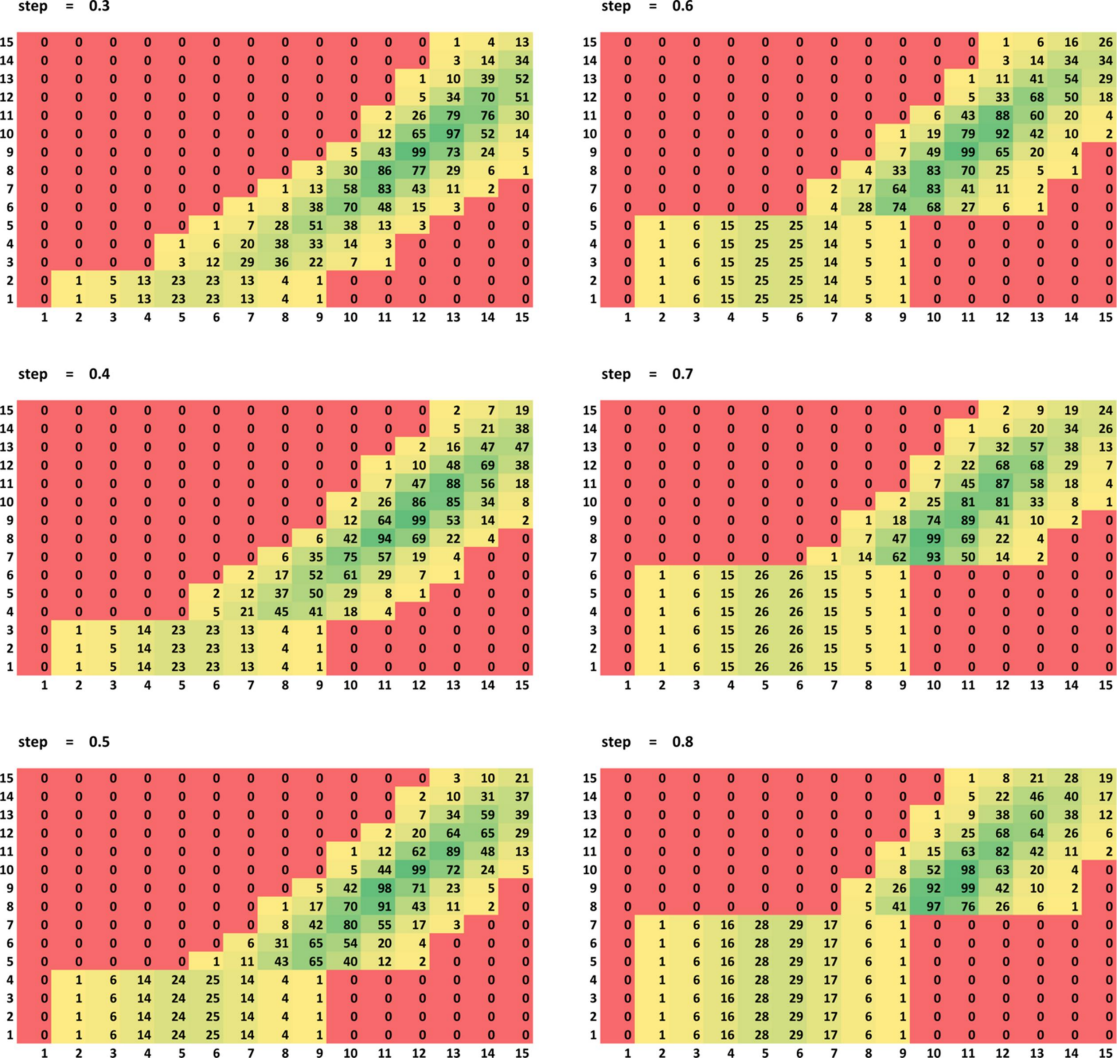
The distribution *P*(*r*_solv_, *r*_shrink_, *d*_grid_) × 10^2^ of the variation in 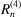 with respect to the best value 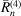 for all combinations of the mask parameters. The values on the axes are *r*_solv_ × 10 (horizontal) and *r*_shrink_ × 10 (vertical). High *P* values correspond to the (*r*_solv_, *r*_shrink_, *d*_grid_) combinations giving 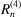 close to the minimum best value of 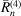 . The colour scheme indicates the *P*-function value ranges: red (*P* < 0.01), yellow (0.01 ≤ *P* < 0.2), light green (0.2 ≤ *P* < 0.9) and dark green (*P* ≥ 0.9).

Fig. 2 ▸ shows the results with ɛ_*R*_ = 0.002 and *C* = 0.5. As expected, the distribution shows that mask shrinking does not impact the results when *r*_shrink_ < *d*_grid_ (rectangular bottom-left regions). Also, the distribution shows a clear nearly linear correlation between *r*_solv_ and *r*_shrink_, giving preferable values roughly at the line *r*_solv_ − *r*_shrink_ ≃ 0.2 Å for each grid step. Finally, it shows that the optimal radii (*r*_solv_, *r*_shrink_) cluster in the range (1.1 ± 0.1 Å, 0.9 ± 0.1 Å).

As expected, increasing the grid step makes 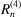 worse. In agreement with the first test (Fig. 1 ▸), most frequently the lowest 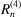 occurred for a grid step size of 0.3–0.4 Å (*P* values up to 0.99). Consequently, a step of 0.4 Å may be considered as a candidate for the most accurate calculations since a smaller step of 0.3 Å does not significantly improve the *R* factors while leading to an increased computational time. Using a grid with step sizes of 0.5–0.6 Å makes it possible for 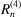 to be close to 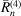 , indicated by high values of the function *P*(*r*_solv_, *r*_shrink_, *d*_grid_) in the range 0.91–0.93. Increasing the step further reduces the maximum *P*-function value to 0.83. Since a larger grid step is preferable to reduce the computing time, *d*_grid_ = 0.6 Å is a good potential compromise candidate for the universal value.

The values of the radii (*r*_solv_, *r*_shrink_) leading to the minimum 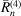 value in (6) also varied only slightly over the trial grid step sizes. This provided a relatively small number of tentative combinations (*r*_solv_, *r*_shrink_, *d*_grid_) to identify the optimal ones which we denote 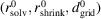 .

### 3.3. Optimal set of parameters

The analysis described in Sections 3.1–3.2 results in a range of (*r*_solv_, *r*_shrink_, *d*_grid_) parameters minimizing *R* ^(4)^ on average for all test models. These values, however, do not necessarily lead to the lowest *R* ^(4)^ for a particular model.

Next, we can ask which of these combinations, if any, lead to a value of 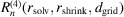 that is larger than, and by how much, the lowest value of 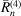 in (6) for what fraction of structures. To answer this question, we calculate the fraction *p*(Δ*R*) of the structures with the difference

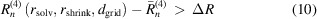

for different Δ*R* values. The expected solution corresponds to the minimum of the *p*(Δ*R*) function. For the sake of completeness, we calculated *p*(Δ*R*) for all triplet values of the parameters considered above.

**Figure 3:**
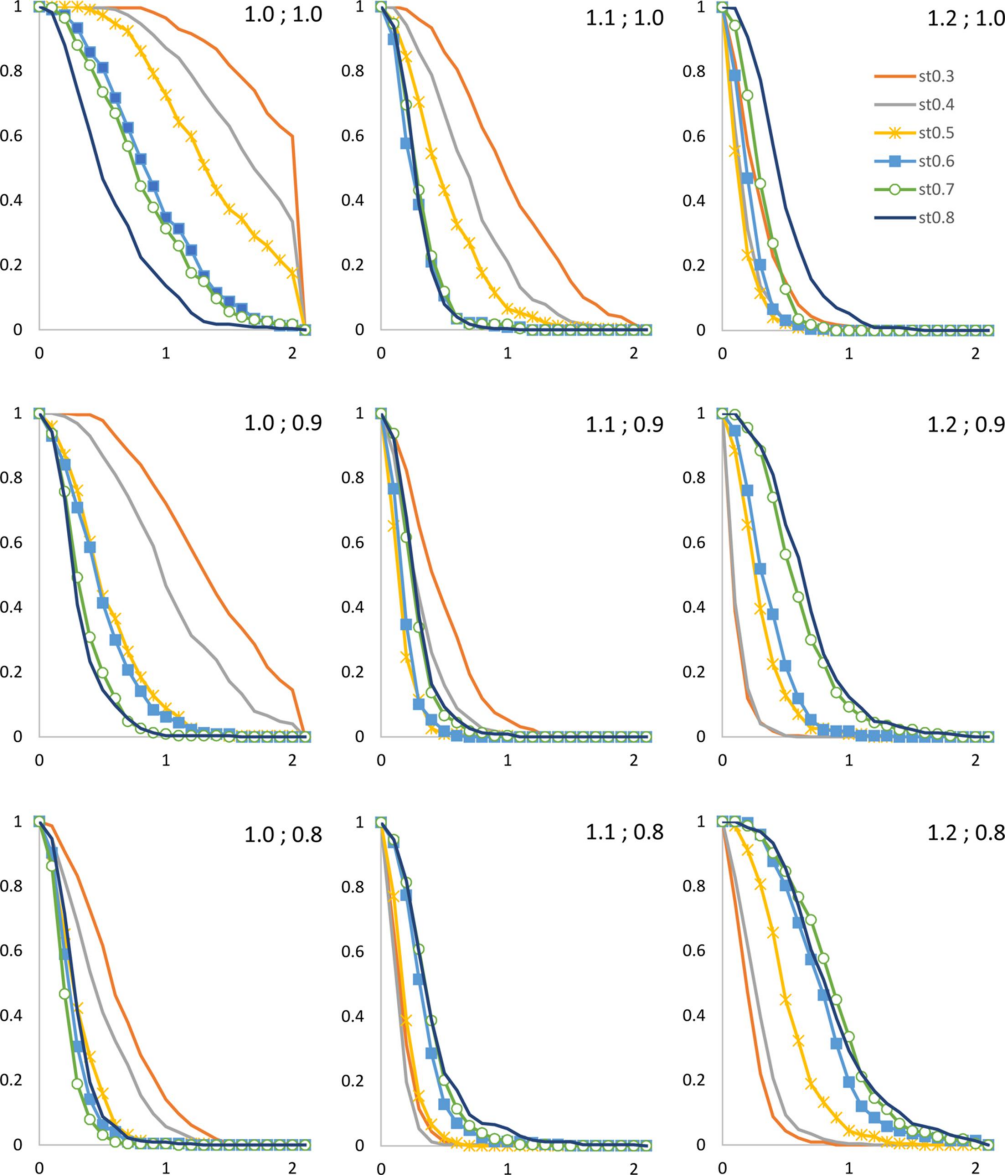
The fraction of the models that satisfy 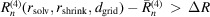 shown as a function of Δ*R*. The colour scheme for the grid step is the same for all plots and is given in the top right plot. The values of 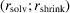 are indicated in each plot.

The combination (*r*_solv_ = 1.0 Å, *r*_shrink_ = 1.0 Å) gives poor results for all grid step sizes and the combination (*r*_solv_ = 1.2 Å, *r*_shrink_ = 0.8 Å) gives results acceptable only for very small grid steps, *d*_grid_ = 0.3–0.4 Å (Fig. 3 ▸). The best results are observed for the sets of *r*_solv_, *r*_shrink_ with *r*_solv_ − *r*_shrink_ = 0.2 Å, with a slight preference for the combination (*r*_solv_ = 1.1 Å, *r*_shrink_ = 0.9 Å, *d*_grid_ = 0.6 Å). Here, 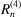 increased by less than 0.5% for all structures of the search set except one, for which this value was below 0.6%. The same plots (Fig. 3 ▸) indicate that if more accurate calculations are required, then the combination (*r*_solv_ = 1.1 Å, *r*_shrink_ = 0.8 Å, *d*_grid_ = 0.4 Å) is optimal. However, using this finer grid step would lead to a nearly fourfold increase in the number of grid points and, consequently, in the number of computational operations required. Conversely, for a very large structure, if a coarser grid is acceptable, a possible combination would be (*r*_solv_ = 1.0 Å, *r*_shrink_ = 0.8 Å, *d*_grid_ = 0.7 Å), resulting in only a slight increase in overall *R* factors.

### 3.4. New versus old mask calculation parameters

Finally, for each model from the complete data set, we analysed how much the *R* ^(4)^ and the *R* factor calculated using all reflection data change if the new mask calculation parameter values ( 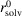 = 1.1 Å, 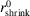 = 0.9 Å, 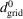 = 0.6 Å) are used instead of the values of (*r*_solv_ = 1.1 Å, *r*_shrink_ = 1.0 Å, *d*_grid_ = *d*_min_/4) used previously.

**Figure 4:**
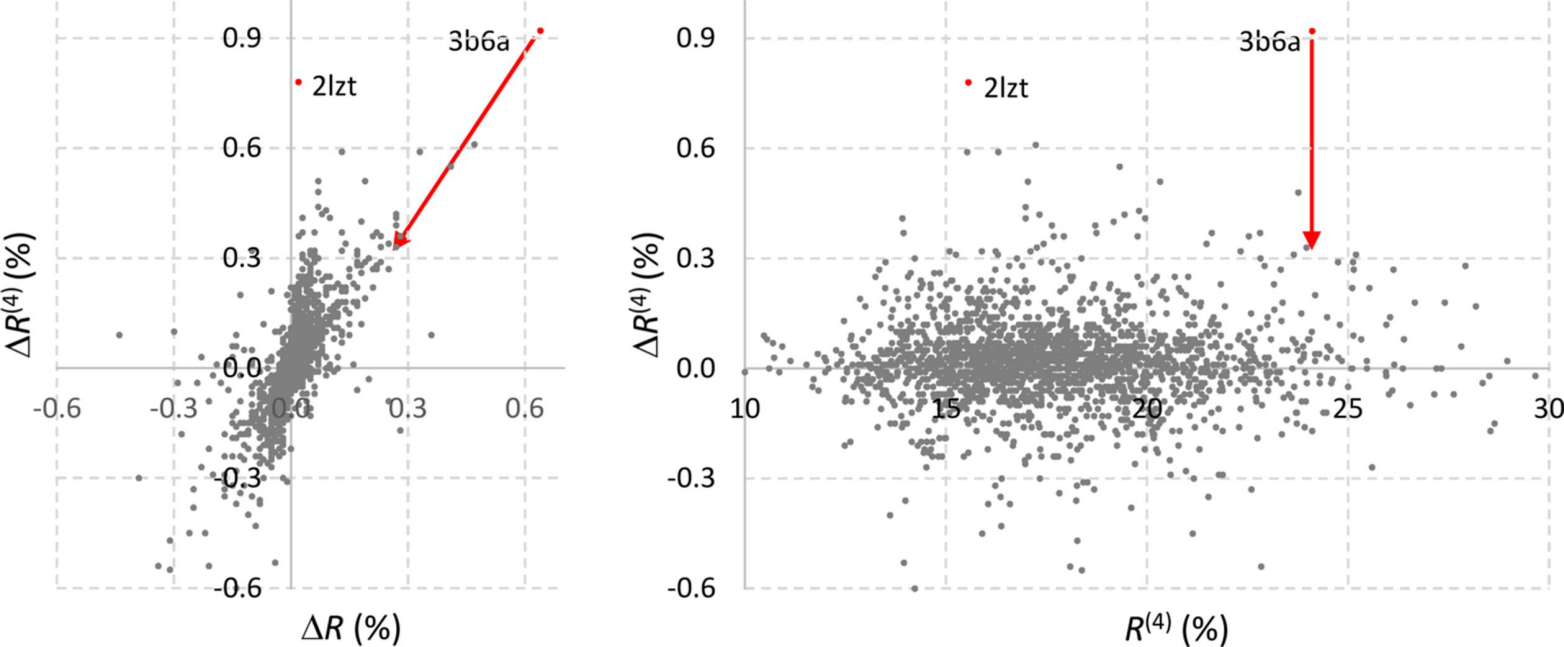
The variation in Δ*R* ^(4)^ calculated for the test set of 2077 models when moving from the conventional set of mask parameters to the selected set 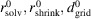 of optimal values. Each point corresponds to an individual model. The plots show the distribution of the variation in Δ*R* ^(4)^ (vertical) versus (left) the variation Δ*R* in the overall *R* factor calculated for the whole set of reflections and (right) the *R* ^(4)^ value itself. For the model of 3b6a (indicated by red arrows) the values (0.64; 0.92) become (0.26; 0.36) after the removal of isolated small-volume regions appearing in the new mask.

Fig. 4 ▸ shows that 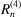 varies little and typically remains within ±0.3% for most structures, with the exception of a few cases where it varies within ±0.5%. We consider these variations negligible. As expected, *R*_*n*_ changes even less than 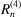 , since the bulk-solvent contribution vanishes beyond 3–4 Å resolution.

The only notable outlier is PDB entry 3b6a (Willems *et al.*, 2008 ▸), for which 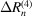 = 0.92% (Δ*R_n_* = 0.64%). This structure was solved at a resolution of *d*_min_ = 3.0 Å, which means that the original algorithm used a mask with a grid step size of 0.75 Å, coarser than the proposed 0.6 Å. This seemingly counterintuitive result can be rationalized as follows. The bulk-solvent mask typically consists of a large region and several (often many) smaller isolated regions (Afonine *et al.*, 2024 ▸). These small regions are typically cavities inside the protein or computational artefacts. The number and size of such regions vary based upon the choice of mask parameters (*r*_solv_, *r*_shrink_, *d*_grid_). With a step size 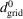 = 0.6 Å, the mask for 3b6a contains about 20 isolated small regions incapable of containing even a single disordered water molecule. Excluding these regions from the bulk-solvent mask reduces 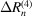 and Δ*R*_*n*_ to 0.36% and 0.26%, respectively, suggesting that these regions are computational artefacts.

## 4. Conclusions

The choice of mask parameters for the flat bulk-solvent model, *i.e.* solvent and mask shrinkage radii and the grid sampling step size, affects both the accuracy of the fit between model and data at medium to low resolution and the speed of the calculations. Since accounting for the bulk solvent typically occurs in crystallographic calculations that involve atomic model and reflection data, from simple operations like *R*-factor or map calculation to complex procedures like model refinement and building, the computational efficiency of this step is critical. The parameters governing the speed and accuracy of the flat bulk-solvent model are the solvent radius *r*_solv_, the mask shrinkage radius *r*_shrink_ and the grid step *d*_grid_ for the mask sampling. When this model was introduced (Jiang & Brünger, 1994 ▸) the choice of values for these parameters, of *r*_solv_ = 1.0 Å, *r*_shrink_ = 1.1 Å and *d*_grid_ = (*d*_min_/4) Å, was based on only two study cases at medium resolution (around 2 Å). A decade later, this choice was revisited for low-resolution cases by Rees *et al.* (2005 ▸), resulting in the suggestion that much finer steps are needed at these resolutions. While these finer steps, calculated as a fraction of *d*_min_, address the problem of accuracy for low-resolution models, they in turn create the problem of substantially increasing the computational time for higher-resolution cases. A universal resolution-independent choice of mask calculation parameters is therefore highly desirable, and we here show that this is possible. A systematic study across hundreds of models from the PDB performed in this work reveals an optimal choice of these parameters to be 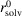 = 1.1 Å, 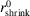 = 0.9 Å and 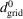 = 0.6 Å. Validation of this choice with a much larger test set of models shows that these values are broadly applicable. In the last stages of refinement, a finer grid with a step *d*_grid_ = 0.4 Å and radii *r*_solv_ = 1.1 Å, *r*_shrink_ = 0.8 Å may possibly be used to improve the results further. The parameters described here are implemented in *CCTBX* and are used in the *Phenix* suite (Liebschner *et al.*, 2019 ▸), where applicable, starting from Version 1.20rc4-4425.

## Appendix A Discrete Fourier transform and Fourier coefficients

For a periodic function *f*(*x*) of a single variable with period *a* = 1, the integral Fourier transform results in an infinite number of Fourier coefficients **F**(*h*), where *h* is an integer. The infinite Fourier series defined by these coefficients converges to the function *f*(*x*). The sharper the function, the faster the convergence is (we do not specify the formal, mathematically strict, conditions of convergence when studying smooth functions like density distributions). When such a function is sampled on a regular grid with *N* points per interval, using the obvious equation for integer *m*

gives the convergent Fourier series on the grid nodes *x*_*n*_ = *n*/*N* which can be expressed as

Here *H* is any integer number, and convergence of the original Fourier series proves convergence of each internal series in the right-hand expression of (12). In other words, given *N* real numbers on a regular grid, the discrete Fourier transform results in a set of values

which possess Hermitian symmetry. There are only *N*/2 independent values since, according to their definition in (13), **F**_grid_(*h*) are periodic and so **F**_grid_(*h*) = **F**_grid_(*h* + *N*) for every *h*. Usually, **F**_grid_(*h*) taken with the consecutive indices are used as approximations to **F**(*h*). In some applications, the range 0 ≤ *h* < *N* can be chosen. However, since the Fourier coefficients **F**(*h*) for convergent series generally decrease with |*h*|, it is more practical to choose −*N*/2 < *h* ≤ *N*/2, which results in a smaller |**F**_grid_(*h*) − **F**(*h*)| difference.
